# Impact of COVID-19 Pandemic on Quality of Life of Type II Diabetes Patients With Periodontitis

**DOI:** 10.3389/froh.2021.682219

**Published:** 2021-06-04

**Authors:** Alicia Morales, Camila Corral-Nuñez, Carolina Galaz, Leslie Henríquez, María Mery, Cesar Mesa, Franz Strauss, Franco Cavalla, Mauricio Baeza, Francisca Valenzuela-Villarroel, Jorge Gamonal

**Affiliations:** ^1^Faculty of Dentistry, Center for Surveillance and Epidemiology of Oral Diseases, University of Chile, Santiago, Chile; ^2^Department of Conservative Dentistry, Faculty of Dentistry, University of Chile, Santiago, Chile; ^3^Department of Restorative Dentistry, Faculty of Dentistry, University of Chile, Santiago, Chile; ^4^Clinic of Reconstructive Dentistry, Center of Dental Medicine, University of Zurich, Zurich, Switzerland; ^5^Department of Oral Biology, University Clinic of Dentistry, Medical University of Vienna, Vienna, Austria; ^6^Faculty of Medicine, University of Chile, Santiago, Chile

**Keywords:** oral health-related quality of life, SARS-CoV2 (COVID- 19), remote monitoring, teledentistry, periodontitis, diabetes mellitus

## Abstract

**Background:** Confinement due to the COVID-19 pandemic has made dental treatments impossible in Chile and many other countries, including diabetic patients with periodontitis. The aim of the present study was to evaluate the impact of periodontal therapy in terms of oral health-related quality of life (OHRQoL) during the COVID-19 pandemic in a cohort of diabetic patients with periodontitis.

**Material and Methods:** Thirty-eight diabetic patients with stage III-IV periodontitis, enrolled for periodontal therapy, were screened. Periodontal clinical parameters including clinical attachment loss (CAL), probing pocket depth (PPD) and bleeding on probing (BOP) as well as glycated hemoglobin (HbA1c) were evaluated at baseline and 3 months follow-up prior the pandemic. The OHRQoL changes by means of Oral Health Impact Profile (OHIP-14) and a self-reported oral health questionnaire were assessed at baseline (prior pandemic) and during the pandemic via telemonitoring.

**Results:** Thirty-one patients received non-surgical periodontal therapy prior to the pandemic. Out of the 31 patients, four died due to COVID-19 resulting in 27 patients available for telemonitoring at the time of the pandemic. Periodontal therapy significantly improved CAL, PPD and BOP (*p* < 0.05) but not HbA1c (*p* > 0.05) between baseline and 3 months follow-up pior to the pandemic. Total OHIP-14 scores significantly improved between baseline and the middle of pandemic (intragroup comparison *p* = 0.00411). In particular, OHIP-14 scores related to the “Physical pain” (intragroup comparison *p* = 0.04) and “Psychological disability” (intragroup comparison *p* = 0.00) significantly improved between baseline and the middle of pandemic.

**Conclusions:** In diabetic type II patients with periodontitis periodontal therapy tends to improve the oral health-related quality of life despite the COVID-19 pandemic.

## Introduction

Diabetes mellitus is a non-communicable disease (NCD) [1] that affects 9.3% of the world population [2] while in Chile, affects about 12% of the population [3]. Its treatment is included in the National Cardiovascular Health Program [4] and the main aim is to improve the metabolic control thereby preventing chronic microangiopathic complications and cardiovascular diseases [5].

Periodontitis, on the other hand, is also considered an inflammatory NCD. Periodontitis is associated with the presence of a dysbiotic biofilm triggering a progressive destruction of the periodontal tissues that may eventually lead to tooth loss [6]. Severe periodontitis affects about 10% of the global population [7]. In Chile, periodontitis affects about 85% of Chilean adults between 35 and 44 years [8] and almost 100% of individuals older than 35 show some tissue attachment loss [9].

Diabetes and periodontitis share the same risk factors [10], and their bidirectional pathogenic association has been widely documented [11]. Clinical studies indicate that diabetics are more likely to have periodontitis [12] and to suffer more severe stages [13]. Moreover, periodontitis in patients with diabetes can adversely affect the glycemic control thereby increasing the risk of other complications, such as retinopathy, and cardiovascular disease [14–16]. Therefore, international organizations have suggested a comprehensive oral health care and comanagement of diabetes and periodontitis [17, 18].

Oral health, from a biopsychosocial perspective, is an integral part of general health capable of generating social, economic and psychological consequences. In other words, oral health can affect individual's quality of life. In fact, Oral health-related quality of life (OHRQoL) is recognized by the World Health Organization (WHO) as an important segment in the Global Oral Health Program [19]. Available evidence suggests that periodontitis and diabetes mellitus have a negative impact on Oral Health-related Quality of Life (OHRQoL) [20, 21]. Hence, it becomes fundamental to measure the impact of oral health related problems on the quality of life. Clinical indicators alone are not capable of representing the expectations, satisfactions or well-being of the patients, and how they cope with their daily life activities [22].

In March 2020, the WHO declared a global pandemic due to the coronavirus (COVID-19) [23]. The transmission of the virus causing this disease occurs by direct or indirect contact with droplets from the airway and respiratory secretions of infected patients [24]. Dental care has become, thus, a potential risk of contagion due to the proximity with the patient and the frequent generation of aerosols in clinical procedures. Indeed, the WHO recommended to postpone any routine non-urgent oral health care, including oral health check-ups and dental cleanings – until a reduction in transmission rates of COVID-19 [25]. As a result, many patients suspended and postponed their dental treatments.

Diabetics have a higher risk of severe COVID-19 [26] and greater probability of worse prognosis and mortality, most likely due to the concurring effect of other comorbidities. Even though periodontal therapy can be considered a contagious risk situation in diabetics, their oral health and glycemic control has to be maintained. However, the impact of periodontal therapy on OHRQoL during the pandemic remains largely unknown. Consequently, it seems reasonable and clinically relevant to explore the OHRQoL during the pandemic in patients that had received periodontal treatment prior to the pandemic.

The aim of the present study was, therefore, to evaluate the impact of periodontal therapy in terms of oral health-related quality of life (OHRQoL) during the COVID-19 pandemic in a cohort of diabetic II patients with periodontitis.

## Materials and Methods

### Study Design

The protocol of this study was revised and approved by the Ethics Committee of the “Metropolitano Occidente” Health Service (Decision number: 21/2018). The present study was conducted within the legal framework for studies in humans in Chile, and in accordance with the Declaration of Helsinki of 1975, revised in 2000. The protocol of the study was explained to all patients, and an informed consent was obtained after the explanation of the purpose, nature, risks and benefits of participating in this study.

### Subject Population and Clinical Criteria for Inclusion and Exclusion

The study population was composed of adults who were in the follow-up stage of the Cardiovascular Health Program at the Doctor Steeger Primary Care Center in Cerro Navia, Santiago de Chile. From the database of this program, volunteers were selected through a non-probability convenience sampling.

Inclusion criteria were: patients of the Cardiovascular Health Program in the follow-up stage (control) diagnosed with type 2 diabetes mellitus, presenting a glycated hemoglobin level (HbA1c) ≥7% (in the last 6 months), and with a diagnosis of periodontitis according to the 2017 Classification of Periodontal and Peri-Implant Diseases and Conditions [27, 28].

Exclusion criteria were: patients who had received periodontal treatment in the last year, females in pregnancy or lactation.

Periodontal clinical parameters were evaluated at six sites in all teeth, excluding third molars. These parameters included probing pocket depth (PPD), dichotomous measurements of supragingival plaque accumulation (PI), and bleeding on probing (BOP) at the base of the crevice. Clinical attachment loss (CAL) was determined using the distance from the cement-enamel junction (CEJ) to the free gingival margin (FGM) and the distance from the FGM to the bottom of the pocket/sulcus. From these two measurements, CAL (distance from the CEJ to the bottom of pocket/sulcus) was calculated. The assessment of the periodontal supporting tissue status was made with a first-generation manual periodontal probe (UNC-15, Hu Friedy Mfg. Co. Inc., Chicago, IL, U.S.A.). At the time of recording depths, if necessary, measurements were rounded to the nearest whole millimeter. One calibrated examiner performed the evaluations and measurements of all patients. Calibration training was performed within successive days during which a group of 10 volunteers were examined. All examinations were repeated until an acceptable consistency was achieved, which was determined by an intra-class correlation coefficient of 0.80.

The levels of HbA1c were measured for all patients at baseline and at 3 months follow-up.

The Oral Health Impact Profile (OHIP)-14 questionnaire was administered to all the participants at baseline and during the pandemic via telemonitoring (due to the confinement). OHIP-14 consists of 14 internationally validated questions [29]. In brief, the questionnaire is subdivided in seven dimensions that comprise the sum of the scores of the different questions. The “Functional limitations” dimension considers the following questions: (1) Have you felt that your breath has worsened because of problems with your teeth? and (2) Have you felt that your digestion has worsened because of problems with your teeth, mouth or denture? The “Physical pain” dimension considers the following questions: (3) Have you had sensitive teeth, for example due to cold food or drinks? and (4) Have you had toothache? The “Psychological discomfort” dimension considers the following questions: (5) “Have you felt totally unhappy because of dental problems?” and (6) “Have you felt dissatisfied with the appearance of your teeth, mouth or denture?” The “Physical disability” dimension includes the following questions: (7) “Has your speech been unclear because of problems with your teeth, mouth or denture?” and (8) “Have people misunderstood some of your words because of problems with your teeth, mouth or denture?” The questions (9) “Has your sleep been interrupted because of problems with your teeth, mouth or denture?” and (10) “Have you been upset because of problems with your teeth, mouth or denture?” are analyzed in the “Psychological disability” dimension. The questions (11) “Have you been less tolerant of your spouse or family because of problems with your teeth, mouth or denture?” and (12) “Have you had difficulty doing your usual job because of problems with your teeth, mouth or denture?” are included in the “Social disability” dimension. The “Handicap” dimension includes the questions (13) “Have you been totally unable to function because of problems with your teeth, mouth or denture?” and (14) “Have you been unable to work to your full capacity because of problems with your teeth, mouth or denture?”. Each question has five possible answers encoded with a score (never = 0; almost never = 1; occasionally = 2; frequently = 3; always = 4). The score of each dimension corresponds to the sum of the scores of the answers to the questions in the dimension. The total OHIP-14 score corresponds to the sum of all answers of the questionnaire. Therefore, the higher the score obtained, the worse the assessment of the oral health-related quality of life.

In addition, a self-reported oral health questionnaire was administered to all the patients during the confinement period [30, 31].

### Intervention

Prior to the pandemic and confinement all articipants underwent non-surgical therapy involving scaling and root planing (SRP) with 1 week-intervals in between up to 4–6 sessions. SRP was performed using an ultrasonic scaler (Cavitron, Dentsply, York, PA, U.S.A) and hand instruments (Hu Friedy Mfg. Co. Inc., Chicago, IL, U.S.A.). Recall visits were performed at 3 months follow-up right before the pandemic.

### Outcome Variables

The primary variable was the change in QHRQoL between baseline and the pandemic. It was analyzed by the score obtained in each dimension of the OHIP-14 and by the total score. The secondary variables were the changes in CAL, PPD, BOP and PI as well as the levels of glycated hemoglobin. Sub-analyses were performed on these outcome variables, taking into account the initial PPD. A pocket was considered as shallow if its initial PPD was ≤ 3 mm, as moderate if its initial PPD was between 4 and 6 mm and as deep if it was ≥7 mm. The percentage of sites, teeth and individuals with a PPD ≥5 mm, ≥6 mm y ≥7 mm was also analyzed, as the percentage of sites with “pocket closure,” defined as the percentage of sites going from PPD ≥4 mm to PPD ≤ 3 mm at 3- and 12-months follow-up [32]. Finally, the answers of the self-reported oral health questionnaire were also analyzed.

### Statistical Analysis

For all statistical evaluations, the patient was the unit of measurement. OHRQoL and periodontal clinical parameters were analyzed for all patients.

Quantitative data were recorded as the mean value ± standard deviation. The scores of the OHIP-14 were reported using the median (interquartile range) and the answers of the self-reported questionnaire were reported in percentage.

The Shapiro-Wilk test was used to evaluate the compliance of parameters with the normal distribution. The Wilcoxon and Student test were used for paired samples. The Fisher's exact test was used for the analysis of categorical variables. The statistical significance was set at *p* < 0.05. The statistical analysis was performed using a statistical package (STATA 16. StataCorp, College Station, TX, U.S.A.).

## Results

Out of the 38 patients, 31 received periodontal therapy, and four of them died due to COVID-19 resulting in 27 patients available for telemonitoring. From these, 77.8% were females; the mean age amounted to 65.3 ± 9.0 years and 37.0% were smokers. The mean number of remaining teeth was 15.3 ± 7.0 and 46.2% of the participants used removable prosthesis. On average, each participant underwent 12 sessions of telemonitoring. At baseline, 92.6% of the patients presented a stage IV periodontitis and an HbA1c level of 8.2 ± 1.7%, prior the pandemic (Table 1).

**Table 1:** Baseline data of treated patients, prior pandemic.

	**Treated group (*n* = 27)**
Age (years)	65.3 ± 9.0
Gender (M/F)	22.2/77.8
Smokers	37.0
HbA1c	8.2 ± 1.7
Dental prosthesis	46.2
Number remaining teeth	15.3 ± 7.0
**Periodontal diagnosis**	
Periodontitis stage III localized grade B	3.7
Periodontitis stage IV generalized grade B	3.7
Periodontitis stage IV localized grade B	7.4
Periodontitis stage IV generalized grade C	85.2

*Data presented as mean ± standard deviation or number (%)*.

The median OHIP-14 scores amounted to 9 at baseline and to 3 during the pandemic, with significant differences between both time points (intragroup comparison *p* = 0.00411) indicating an improvement in the quality of life (Table 2). Moreover, the OHIP-14 scores related to the “Physical pain” (intragroup comparison *p* = 0.04552) and “Psychological disability” (intragroup comparison *p* = 0.00691) dimensions significantly improved between baseline and the pandemic (Table 2).

**Table 2:** Baseline and pandemic OHIP-14Sp score in treated patients [median (interquartile range)].

	**Treated group (*n* = 27)**
**Functional limitations**	
Basal	2 (0)
Pandemic	0 (2)
*p*-value	0.6705
**Physical pain**	
Basal	2 (2)
Pandemic	0 (3)
*p*-value	**0.0455** ^**1**^
**Psychological discomfort**	
Basal	3 (6)
Pandemic	0 (4)
*p*-value	0.0625
**Physical disability**	
Basal	0 (2)
Pandemic	0 (2)
*p*-value	0.9009
**Psychological disability**	
Basal	2 (3)
Pandemic	0 (2)
*p*-value	**0.0069** ^**1**^
**Social disability**	
Basal	0 (0)
Pandemic	0 (0)
*p*-value	0.6250
**Handicap**	
Basal	0 (0)
Pandemic	0 (0)
*p*-value	0.2500
**Global**	
Basal	9 (13)
Pandemic	3 (8)
*p*-value	**0.0041** ^**1**^

1 *Intra- group comparison by Wilcoxon signed rank test. Statistical significance in bold test (p < 0.05)*.

The answers to the self-reported questionnaire are displayed in Table 3. More than 50% of the individuals considered their oral health as Very good/Good. At the same time, 70.4% of the patients considered that their oral health was maintained during the confinement. Regarding the increase of tooth mobility, gingival recession, gum pain, and gingival bleeding, most of the individuals reported that they “did not” present these problems (*p* > 0.05). More than half of the patients thought that some tooth had a problem during the confinement. During the confinement, no patient visited the and no teeth were lost because of mobility or pain (data not shown).

**Table 3:** Self-reported screening questionnaire, frequency of responses to the self-reported questions.

**Self- reported questions and answers**	**Treated group (*n* = 27)**
Overall, how would you rate the health of your teeth and gums during confinement:	
Very good/good	51.9%
Fair	33.3%
Poor/very poor	14.8%
Don't know/don't answer	0.0%
If you compare your current oral health with what it was before confinement, you consider that this is	
Better	3.7%
Remained	70.4%
Worse	25.9%
Don't know/don't answer	0.0%
Do you think you might have gum disease?	
Yes	29.6%
No	63.0%
Don't know/don't answer	7.4%
Have you noticed that some of your teeth have become loose on their own, without having suffered a trauma during this period of confinement??	
Yes	29.6%
No	70.4%
Don't know/don't answer	0.0%
Have you noticed that your teeth are longer or that you have receding gums during this period of confinement??	
Yes	18.5%
No	77.8%
Don't know/don't answer	3.7%
Have you noticed that you see the roots of several of your teeth during this period of confinement??	
Yes	3.7%
No	92.6%
Don't know/don't answer	3.7%
Have you felt pain in your gums during this period of confinement?	
Yes	37.0%
No	63.0%
Don't know/don't answer	0.0%
Have you felt pain in your teeth during this period of confinement?	
Yes	25.9%
No	74.1%
Don't know/don't answer	0.0%
Have you noticed that any of your teeth have a problem during this period of confinement?	
Yes	55.6%
No	44.4%
Don't know/don't answer	0.0%
Do your gums usually bleed either when brushing or chewing?	
Yes	14.8%
No	85.2%
Don't know/don't answer	0.0%
At this time, do you think you have a dental emergency?	
Yes	7.4%
No	88.9%
Don't know/don't answer	3.7%

Periodontal clinical parameters and levels of HbA1c are shown in Table 4. CAL, PPD, BOP, and PI in moderate and deep sites revealed a significant improvement between baseline 3 months follow-up (prior to the pandemic) along with a decrease in the percentage of sites and teeth with PPD ≥5 mm. HbA1c levels did not significantly decrease after periodontal treatment and prior to the pandemic.

**Table 4:** Clinical parameters and glycated hemoglobin at baseline and 3 months follow-up in treated group (*n* = 27), prior pandemic.

**Parameter**	**Baseline**	**3 months**	***p-*value**
CAL (mm)	4.2 ± 1.2	4.0 ± 1.1	**0.0020**
PPD (mm)	2.8 ± 0.6	2.4 ± 0.5	**0.0001**
BOP (%)	55.6 ± 15.4	33.6 ± 18.8	**0.0001**
PI (%)	77.8 ± 14.5	54.3 ± 19.5	**0.0001**
Moderate sites (CAL)	5.6 ± 1.0	5.0 ± 1.2	**0.0001**
Moderate sites (PPD)	4.3 ± 0.3	3.4 ± 0.7	**0.0001**
Deep sites (CAL)	8.8 ± 1.3	7.1 ± 1.8	**0.0159**
Deep sites (PPD)	7.4 ± 0.5	5.0 ± 2.3	**0.0219**
**Follow- up of moderate pockets detected at baseline (%)**			
PPD ≤ 3 mm		55.1 ± 36.5	–
PPD 4–6 mm		43.6 ± 36.9	–
PPD ≥7 mm		0.0%	–
Follow- up of deep pockets detected at baseline (%)			
PPD ≤ 3 mm		9.3 ± 27.9	–
PPD 4–6 mm		5.5 ± 21.2	–
PPD ≥7 mm		11.1 ± 32.0	–
Pocket closure (%)		54.6 ± 36.5	–
Sites with PPD ≥5 mm (%)	7.2 ± 12.3	4.2 ± 10.1	**0.0002**
Sites with PPD ≥6 mm (%)	2.8 ± 6.8	1.9 ± 6.5	0.0625
Sites with PPD ≥7 mm (%)	1.1 ± 2.9	0.6 ± 2.6	0.1250
Teeth with PPD ≥5 mm (%)	22.7 ± 27.6	14.2 ± 23.5	**0.0002**
Teeth with PPD ≥6 mm (%)	9.2 ± 16.5	5.7 ± 15.0	0.0742
Teeth with PPD ≥7 mm (%)	4.1 ± 9.0	1.9 ± 7.2	0.1250
Patients with PPD ≥5 mm (%)	77.7%	59.2%	0.0625
Patients with PPD ≥6 mm (%)	40.7%	22.2%	0.1797
Patients with PPD ≥7 mm (%)	25.9%	11.1%	0.1250
Glycated hemoglobin (%)	8.2 ± 1.7	8.1 ± 1.4	0.9009

*Intra- group comparison by Wilcoxon signed rank test, Student test and Mc Nemar test. Statistical significance in bold (p < 0.05).*

*Data presented as mean ± standard deviation or number (%). CAL, clinical attachment loss; PPD, probing pocket depth; BOP, bleeding on probing; PI, plaque index*.

## Discussion

The present study in diabetics with periodontitis predominantly revealed that periodontal therapy tends to improve the oral health-related quality of life despite the confinement due to the COVID-19 pandemic.

By using OHIP-14, the present study aimed to evaluate the quality of life following periodontal therapy in diabetics with periodontitis at the time of COVID confinement via telemonitoring. The present study revealed an increase in the OHRQoL between baseline and the COVID confinement, indicated by the overall improvement of the OHIP-14 scores. Moreover, a significant improvement occurred in the dimensions related to “physical pain” and “psychological disability” between baseline and confinement. These improvements in OHRQoL compare well with previous studies where an improvement in OHRQoL was observed after periodontal treatment [20, 33]. Periodontitis has been associated with lower levels of OHRQoL in different populations, with an impact on physical dimensions, such as halitosis and diet, psychological and social, and happiness-related dimensions [34–46], which has also been observed in diabetic patients with periodontitis [47]. Low levels in OHRQoL has been associated with the severity of periodontal disease likely due to a greater clinical attachment loss provoking a higher impact on the functional limitation, physical pain, psychological discomfort, and physical and psychological disability [36, 37, 41, 43, 45, 46]. A recent systematic review revealed that periodontitis reduced the OHRQoL and its impact increased with the severity and extension of the disease [48]. In this regard, periodontal treatment has consistently shown a positive impact on the OHRQoL [49–53] consistent with the present findings. In the present study, the patients presented stage III or IV periodontitis, revealing severe periodontal destruction and tooth loss. Notabty, the non-surgical therapy was able to improve the OHRQoL even though it is just the second step of therapy [54]. These patients require additional treatment steps including surgical interventions and prosthetic rehabilitations which could not be performed due to the pandemic.

OHIP-14 is one of the most common and validated questionnaires to measure oral health-related quality of life [29]. It is based on the conceptual model proposed by D. Locker in 1988 and the WHO [19]. Despite being a short questionnaire, it has proven to be highly reliable, sensitive, accurate and with an adequate cross-cultural consistency. Furthermore, it has been used in both cross-sectional and longitudinal studies. However, its limitations should not be ignored. For example, some studies have indicated that OHIP-14 is not as effective as other questionnaires in detecting the impact of oral disorders in the population, such as the *General Oral Health Assessment Index* (GOHAI). This reduced efficacy of OHIP-14 is most likely explained by type of questions, which are more focused on the psychological and behavioral aspects (and to a lesser extent on functionality, pain and discomfort). In general, OHIP-14 tends to generate a higher percentage of zero scores (floor effect) thus affecting the ability of the questionnaire to detect robust changes. Therefore, its use for the evaluation of the therapeutic interventions has been questioned. Indeed, an improvement in the OHRQoL in those items could be overlooked in subjects who had a zero score at baseline [55, 56].

Periodontal therapy produced a significant attachment gain as wells as a decrease in PPD, BOP and PI. These findings are largely consistent with previous studies [57] on diabetic patients [10]. Nevertheless, a reduction of HbA1c due to the periodontal treatment was not observed. This is contrast to several previous reports revealing a reduction of HbA1c in diabetic patients after periodontal therapy [58–64]. The lack of HbA1c reduction in the present study might be explained by the small sample size as well as the selection bias and other confounding variables such as other comorbidities.

The present study has a number of limitations that should be considered when interpreting the present findings. Firstly, the small sample size that limits the power of the study and the generalization of the present observations. Secondly, the selection bias and other potential sources of bias as well as the presence of other confounding variables — including other comorbidities — that were not addressed in the present report. Finally, it remains unknown to what extent the therapy or the pandemic influenced the present results since an evaluation of OHRQoL after periodontal periodontal and before the pandemic could not be performed. Further studies in larger cohorts are needed to confirm these preliminary findings.

## Conclusions

In diabetic type II patients with periodontitis periodontal therapy tends to improve the oral health-related quality of life despite the COVID-19 pandemic.

## Data Availability Statement

The original contributions presented in the study are included in the article/supplementary material, further inquiries can be directed to the corresponding author/s.

## Ethics Statement

The studies involving human participants were reviewed and approved by Comité Etica, Servicio de Salud Metropolitano Occidente. The patients/participants provided their written informed consent to participate in this study.

## Author Contributions

AM, JG, and FS: conception and design of the study, literature search, analysis and interpretation of data collected, drafting of article and/or critical revision, and final approval and guarantor of manuscript. CG, FV-V, and LH: data collection. MM and CM examined and treated patients while FC and MB monitored the process. All authors contributed to the article and approved the submitted version.

## Conflict of Interest

The authors declare that the research was conducted in the absence of any commercial or financial relationships that could be construed as a potential conflict of interest.
